# Differences in the Mechanical Properties of the Developing Cerebral Cortical Proliferative Zone between Mice and Ferrets at both the Tissue and Single-Cell Levels

**DOI:** 10.3389/fcell.2016.00139

**Published:** 2016-11-25

**Authors:** Arata Nagasaka, Tomoyasu Shinoda, Takumi Kawaue, Makoto Suzuki, Kazuaki Nagayama, Takeo Matsumoto, Naoto Ueno, Ayano Kawaguchi, Takaki Miyata

**Affiliations:** ^1^Department of Anatomy and Cell Biology, Graduate School of Medicine, Nagoya UniversityNagoya, Japan; ^2^Division for Morphogenesis, Department of Developmental Biology, National Institute for Basic BiologyOkazaki, Japan; ^3^Micro-Nano Biomechanics Laboratory, Department of Intelligent Systems Engineering, Ibaraki UniversityHitachi, Japan; ^4^Biomechanics Laboratory, Department of Mechanical Engineering, Nagoya Institute of TechnologyNagoya, Japan

**Keywords:** neuroepithelium, apical surface, elasticity, neural progenitor cell, interkinetic nuclear migration, atomic force microscopy, actomyosin, cell density

## Abstract

Cell-producing events in developing tissues are mechanically dynamic throughout the cell cycle. In many epithelial systems, cells are apicobasally tall, with nuclei and somata that adopt different apicobasal positions because nuclei and somata move in a cell cycle–dependent manner. This movement is apical during G2 phase and basal during G1 phase, whereas mitosis occurs at the apical surface. These movements are collectively referred to as interkinetic nuclear migration, and such epithelia are called “pseudostratified.” The embryonic mammalian cerebral cortical neuroepithelium is a good model for highly pseudostratified epithelia, and we previously found differences between mice and ferrets in both horizontal cellular density (greater in ferrets) and nuclear/somal movements (slower during G2 and faster during G1 in ferrets). These differences suggest that neuroepithelial cells alter their nucleokinetic behavior in response to physical factors that they encounter, which may form the basis for evolutionary transitions toward more abundant brain-cell production from mice to ferrets and primates. To address how mouse and ferret neuroepithelia may differ physically in a quantitative manner, we used atomic force microscopy to determine that the vertical stiffness of their apical surface is greater in ferrets (Young's modulus = 1700 Pa) than in mice (1400 Pa). We systematically analyzed factors underlying the apical-surface stiffness through experiments to pharmacologically inhibit actomyosin or microtubules and to examine recoiling behaviors of the apical surface upon laser ablation and also through electron microscopy to observe adherens junction. We found that although both actomyosin and microtubules are partly responsible for the apical-surface stiffness, the mouse<ferret relationship in the apical-surface stiffness was maintained even in the presence of inhibitors. We also found that the stiffness of single, dissociated neuroepithelial cells is actually greater in mice (720 Pa) than in ferrets (450 Pa). Adherens junction was ultrastructurally comparable between mice and ferrets. These results show that the horizontally denser packing of neuroepithelial cell processes is a major contributor to the increased tissue-level apical stiffness in ferrets, and suggest that tissue-level mechanical properties may be achieved by balancing cellular densification and the physical properties of single cells.

## Introduction

**Figure 1:**
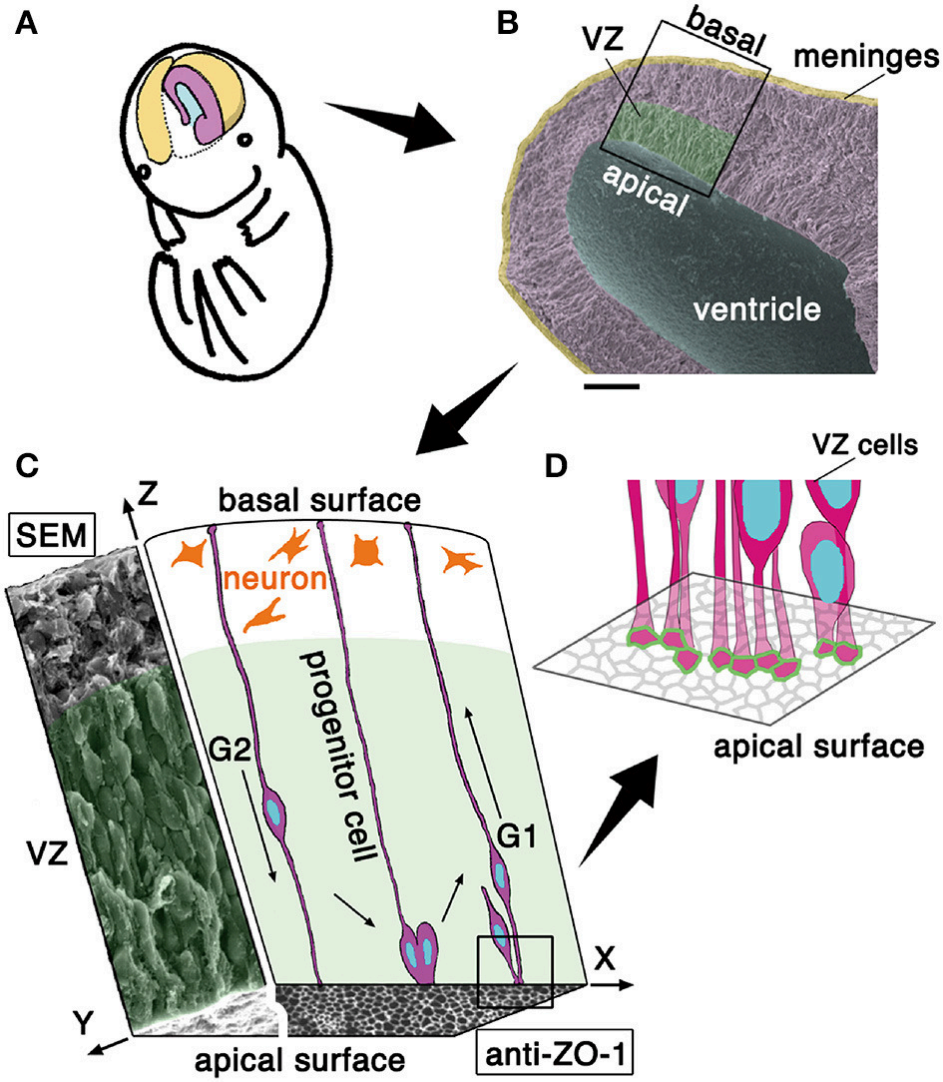
**Schematic representation of the embryonic cerebral wall subjected to the present mechanical measurement. (A)** Illustration of the cerebral hemispheric wall (colored). **(B)** Scanning electron micrograph picture of a cerebral wall isolated from an embryonic day (E) 13 mouse. Scale bar, 100 μm. **(C)** 3D illustration of a portion of the cerebral wall. In the xz plane, the cross-sectional schematic view depicts progenitor cells spanning apicobasally across the wall and neurons accumulating in the outer zone. In the yz plane, the cross-sectional view obtained by scanning electron microscopy (SEM) shows an apparent stratification of cell bodies. The xy plane (an *en face* view of the ventricular or apical surface of the wall) consists of a SEM portion (left) and an immunohistochemically stained portion (right). While the outer neuronal territory is really stratified, the inner progenitor territory, called the ventricular zone, VZ is “pseudostratified,” with each nucleus migrating in a cell cycle–dependent manner within an elongated progenitor cell whose apical endfoot is integrated into the adherens junction meshwork visualized by anti–ZO-1 immunostaining. Note that VZ is filled with somata and cellular processes with no gaps. **(D)** Schematic illustration the apical-most microzone, showing the tangential assembly of the apical processes of VZ cells and the formation of a junctional meshwork (green, corresponding to ZO-1 immunoreactivity in **C**).

The formation of mammalian central nervous system structures (i.e., brains and spinal cord) begins with the emergence of the neuroepithelium, which is derived from the ectoderm, and consists of undifferentiated neural progenitor cells. Cross-sectionally, neuroepithelia (50–100 μm thick in early embryonic mice) are sandwiched by, apically, a fluid-filling space (called the ventricle) and, basally, the mesenchyme-derived component that will differentiate into meninges. As the brain walls thicken (~200 μm, in mid-embryonic mouse cerebrum), a new zone where neurons accumulate in large number is added outside the original neuroepithelial part that is thereafter called the ventricular zone (VZ) (~100 μm) (Figure 1) (Götz and Huttner, 2005; Miyata, 2008; LaMonica et al., 2012; Taverna et al., 2014).

The apical surface of developing brain walls is formed by tangential assembly of the apical endfeet of neuroepithelial or VZ cells, and this assembly can be visualized as meshes immunopositive for molecules enriched in adherens junction, like ZO-1 (Figure 1C) or cadherins (Kosodo et al., 2004). Neuroepithelial and VZ cells are apicobasally elongated, with narrow (<10 μm^2^) apices (Nishizawa et al., 2007, in embryonic mouse cerebrum) (Figure 1C). As in a variety of epithelial cells, the apex of neuroepithelial or VZ cells is contractile in an actomyosin-dependent manner, and the entire apical surface is under tangential tension. This apical-surface contractility thereby bends or curls the walls toward the apical side (Nishimura et al., 2012; Suzuki et al., 2012; Kadoshima et al., 2013; Okamoto et al., 2013, as shown in **Figure 3A**), causing the developing cerebral hemispheric walls to take on a dome-like, apically concave shape (Figures 1A,B). Despite such qualitative understanding of the apical surface's physical property, as well as the potential importance of periventricular mechanical factors in the overall neuroepithelial dynamics (Norden et al., 2009; Kosodo et al., 2011; Leung et al., 2011; Okamoto et al., 2013), quantitative assessments that focus on the elasticity provided by the apical surface and nearby cellular structures have not been made.

The neuroepithelium and the VZ are both pseudostratified, which means that although most of the cells are connected to both apical and basal surfaces of the brain wall to form an epithelial monolayer, their nuclei and somata adopt different apicobasal positions (Figure 1C). The reason for the diffusely staggered nuclear and somal positioning (Figures 1B,C, scanning electron microscopy) is that proliferative cells move their nuclei and somata apically during G2 phase of the cell cycle before they divide at the apical surface and their daughter cells move nuclei/somata basally during G1, using their apicobasally elongated morphology (Figure 1C). These cell-cycle phase-dependent nuclear/somal movements are collectively called interkinetic nuclear migration (INM) (reviewed in Miyata, 2008; Taverna and Huttner, 2010; Kosodo, 2012; Lee and Norden, 2013). The neuroepithelium and the VZ are filled with moving somata and apicobasally oriented processes, with minimal gaps and extracellular spaces (Hinds and Ruffett, 1971).

Analogously to industrial production events in human society that rely heavily on the trafficking or movement of materials and products, INM may contribute to the successful systematic production of cells at the apical surface, thereby supporting overall brain formation (Smart, 1972). Indeed, we recently found that experimental inhibition of basalward INM resulted in nuclear/somal overcrowding on the subapical surface (~30 μm from the surface), which in turn induced the abnormal detachment of proliferative cells from the apical surface and the disruption of histogenesis (Okamoto et al., 2013). This finding suggests that proliferative cells may sense and respond to excessive mechanical stress generated in the subapical space. It also raises the possibility that similar sensing and responses to mechanical factors might also occur under physiological (non-overcrowded) conditions, possibly coordinating heterogeneous nuclear and somal movements (Miyata et al., 2015; Strzyz et al., 2016). We chose to focus on a microzone near (~10 μm from) the apical surface, where multiple mechanical events, such as mitosis, resultant duplication of nuclear and somal flow, as well as apical constriction, take place. We decided that this location was a good candidate for where the sensing of mechanical factors by VZ cells occurs. Given increasing necessity of studying the mechanical control of nervous system development (Franze, 2013), measuring physical parameters at or near the apical surface thus provides a platform for studying previously unexplored aspects of brain development, namely intra-neuroepithelial mechanics and crowd dynamics.

**Figure 2:**
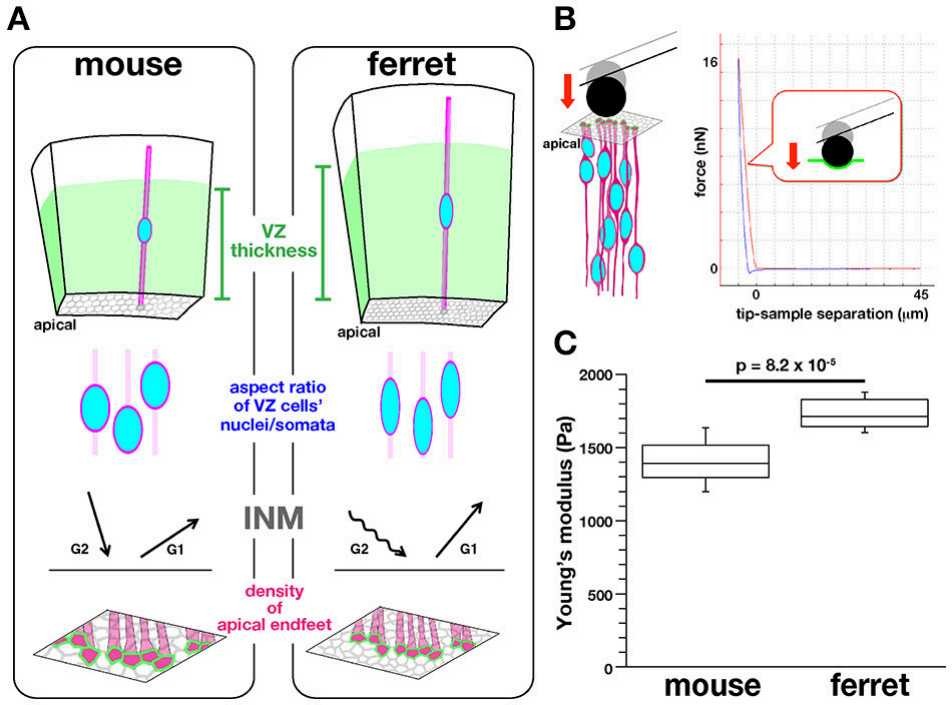
**Background, experimental design, and results of AFM measurements made on the apical surface of cerebral walls**. **(A)** Previously reported mouse–ferret differences in the thickness of the VZ (top), the aspect ratios of the nuclei and somata of VZ cells (second row), the pattern of INM (third row), and the density of VZ cell apices (bottom) (Okamoto et al., 2014). **(B)** Illustration showing AFM indentation measurement on the apical surface of cerebral walls (left) and a typical force–distance curve obtained (right). **(C)** Graph showing that the elastic modulus measured vertically on the apical surface was greater for ferret VZ (*n* = 12) than for mouse VZ, *n* = 11 (*p* = 8.2 × 10^−5^, Mann-Whitney *U*-test).

To establish a technical basis for dissecting the possible links between intra-neuroepithelial nuclear/somal traffic and physical parameters that proliferative cells face in the subapical space, we sought to compare the stiffness on or near the apical surface of the embryonic cerebral VZ between mice and ferrets in a quantitative manner (Figure 2). We had recently found that in ferrets, a microzone containing the apical surface is horizontally denser in terms of the apical process of VZ cells (39.6 apices per 100 μm^2^) than in mice (27.6 apices 100 μm^2^) (Figure 2A), and also that INM behaviors were different between mice and ferrets (i.e., apicalward movement of G2-phase cells' nuclei/somata was slower in ferrets, while basalward movement of early G1-phase cells nuclei/somata was quicker in ferrets than in mice, Figure 2A) (Okamoto et al., 2014). The cerebral cortex of adult ferrets, which has gyri and sulci as in primates including humans, is larger than that of mice, which lack gyri and sulci. Moreover, the total volume of the embryonic cerebral cortical VZ is greater in ferrets than in mice (Figure 2A). The present mouse-ferret comparison will thus provide insights into possible mechanical modulations that have occurred during evolutionary expansion of the neuroepithelium or VZ.

Our measurements using atomic force microscopy revealed that the apical surface of the mouse VZ was stiffer than that of the ferret VZ. We systematically analyzed factors underlying the apical-surface stiffness through experiments to pharmacologically inhibit actomyosin or microtubules and to examine recoiling behaviors of the apical surface upon laser ablation. We found that although both actomyosin and microtubules are partly responsible for the apical-surface stiffness, the greater stiffness of the ferret apical surface compared with mice is largely explained by the denser lateral assembly of apical endfeet in ferrets. We also found that the stiffness of single dissociated VZ cells was greater in mice than in ferrets. Electron microscopy revealed that adherens junction was comparable between the mouse VZ and ferret VZ. Furthermore, non-somal cellular elements (apical processes) showed greater (76% in ferrets and 60% in mice) occupancy than nuclei/somata in the apicalmost zone (~5 μm from the surface). These results provide a solid basis for further biomechanical understanding of mechanisms underlying the development of brains.

## Results

### Apical surface is stiffer in the ferret VZ than in the mouse VZ

Mouse and ferret VZ cells have recently been found to differ in the degree of pseudostratification (i.e., VZ thickness), the nuclear/somal aspect ratio (ferret VZ cells are more slender), the horizontal/tangential packing density (ferret VZ cells are more densely packed), and the INM behaviors (Okamoto et al., 2014) (Figure 2A). To quantitatively determine whether they are under different mechanical conditions on or near the apical surface, we first performed indentation measurement to infer the stiffness using an atomic force microscope (AFM) (Franze, 2011; Iwashita et al., 2014; Gautier et al., 2015). Fresh cerebral walls (200–300 μm thick in mice, 300–400 μm thick in ferrets) were prepared from embryonic mice and ferrets at equivalent developmental stages (E13 in mice and E28–30 in ferrets) (Clancy et al., 2001), and placed on gel-coated dishes with the apical surface facing up. The apical surface was pushed with a spherical bead (20 μm diameter) attached to the tip of a cantilever (with an indentation depth of 5 μm) (Figure 2B). From the force–distance curves obtained subsequently, the elastic (Young's) moduli were determined. As shown in Figure 2C, the elastic modulus obtained vertically on the apical surface of the ferret VZ was significantly greater (1734.3 ± 49.9 Pa, *n* = 12) than that obtained for the mouse VZ (1398.8 ± 99.8 Pa, *n* = 11) (*p* = 8.2 × 10^−5^, Mann-Whitney *U*-test), indicating that the apical surface and the nearby microzone are stiffer in ferrets than in mice.

### Pharmacological studies of apical surface stiffness

**Figure 3:**
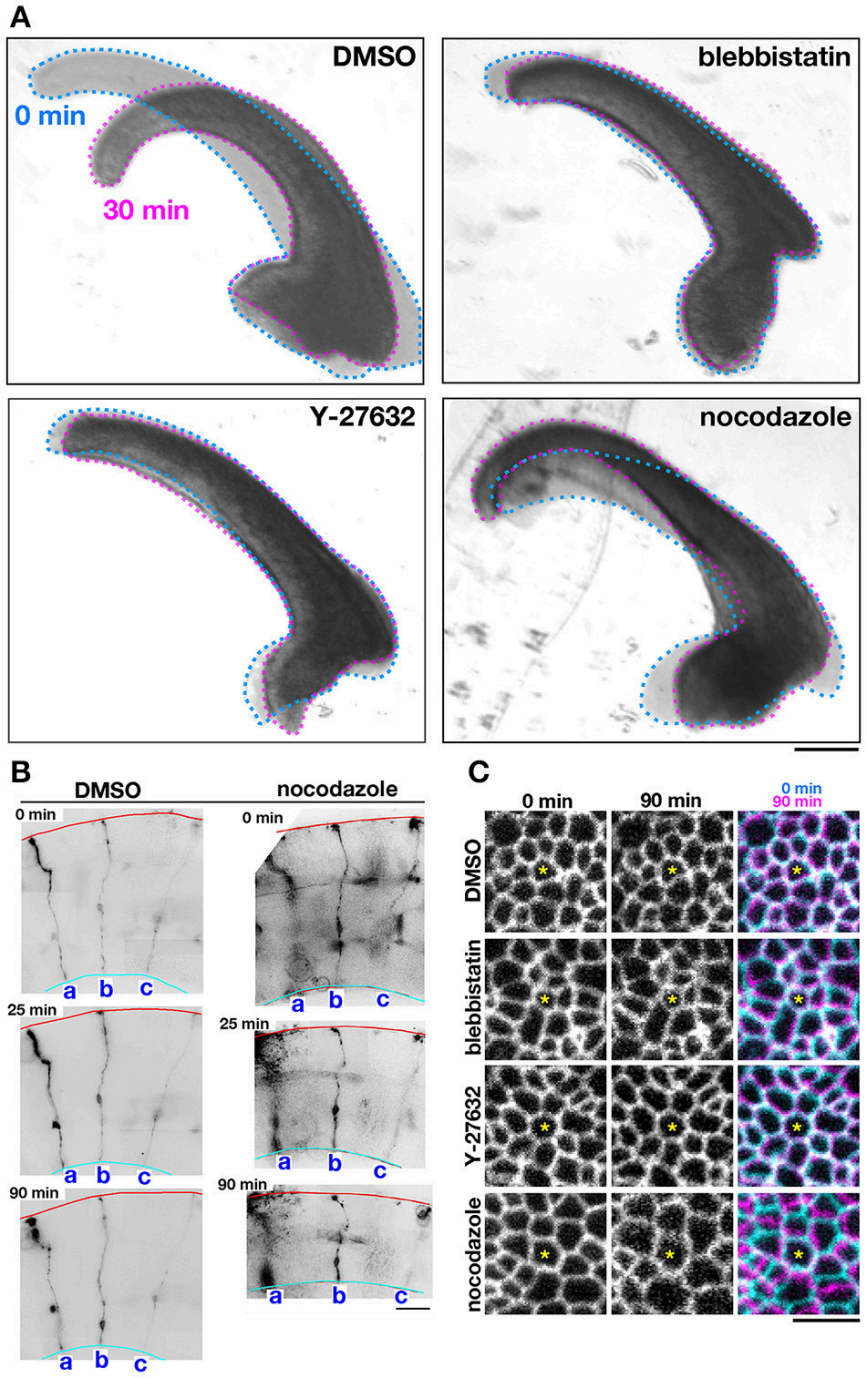
**Effects of pharmacological inhibitors on the mechanical behavior of cerebral walls and apical surface**. **(A)** Photomicrographs showing apical bending/curling of E13 mouse cerebral wall slices exposed to DMSO, blebbistatin, Y-27632, or nocodazole. Note that blebbistatin and Y-27632 clearly blocked bending/curling, and also that nocodazole laterally expanded and thinned cerebral walls, making bending/curling less evident. **(B)** Observation of sparsely DiI-labeled three progenitor cells (a-c) in slices treated with DMSO or nocodazole. In the nocodazole-treated slice, thinning and lateral expansion of cerebral wall was coupled with separation between the tracked progenitors. **(C)** Live monitoring of the apical junction mesh using R26-ZO1-EGFP mice. In each set of panels, one endfoot was used as a reference (center, asterisk). While the size of meshes and the distance between meshes did not change in cerebral walls treated with blebbistatin or Y-27632, the nocodazole-treated apical surface showed enlargement of meshes and lateral separation between meshes. Scale bar, 200 μm in **(A)**, 50 μm in **(B)**, 5 μm in **(C)**.

**Figure 4:**
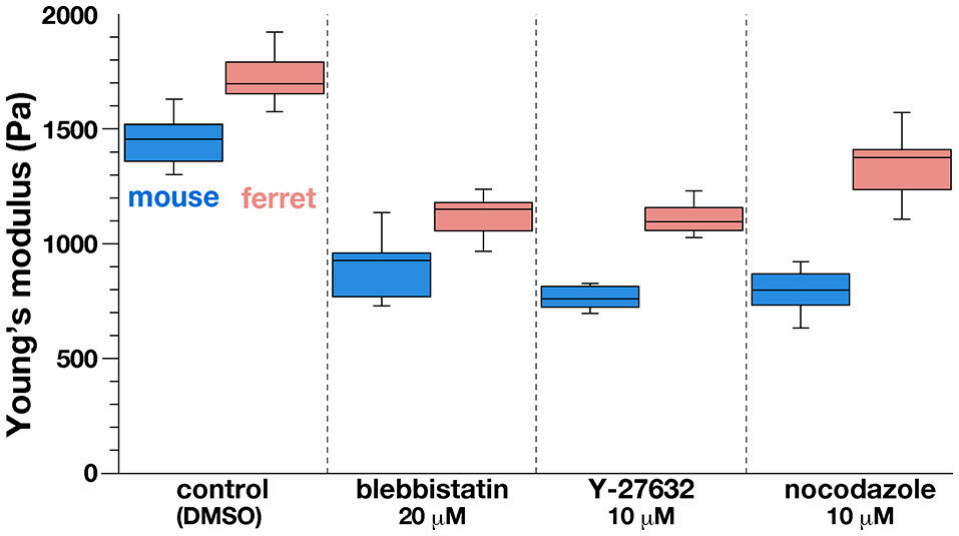
**Graph depicting the results of AFM indentation experiments in the presence of pharmacological inhibitors that affect the apical surface**. Note that the elastic modulus was reduced by all the inhibitors tested in both mice and ferrets, in keeping with the original mouse<ferret relationship.

As a first approach to identifying the basis for the mouse–ferret difference in the elastic modulus at/near the apical surface of VZ, we coupled AFM indentation measurements with pharmacological experiments. Inhibiting myosin with either blebbistatin or Y-27632 (a rho-kinase inhibitor) reduces contractility along the apical surface (Kinoshita et al., 2008), thereby leading to the loss of apical bending/curling of cerebral walls (Figure 3A), although our live observation of the apical surface visualized with ZO1-EGFP before and after blebbistatin or Y-27632 showed that apical mesh size did not change (Figure 3C). Similar actomyosin-dependent bending/curling occurred in ferret cerebral walls (data not shown). Under actomyosin-inhibited conditions, we found that the elastic moduli decreased (by 30–50%) in both mice and ferrets, but significant mouse–ferret differences were observed even in the presence of blebbistatin (*p* = 0.0004) or Y-27632 (*p* = 4.2 × 10^−5^), as in the control (1% DMSO, *p* = 0.0002, Mann-Whitney *U*-test) (Figure 4).

Microtubules play a role in the apicobasal elongation of neuroepithelial cells (Karfunkel, 1971; Miyata and Ogawa, 2007; Suzuki et al., 2012). It is also known that the loss of microtubules and resultant reduction of the neuroepithelial height leads to an increase in the neuroepithelial width (Schoenwolf and Powers, 1987). The addition of nocodazole resulted in thinning and lateral expansion of cerebral walls (Figures 3A,B), with horizontal separation between sporadically fluorescent labeled neural progenitor cells (Figures 3B,C), as well as increased apical mesh size (Figure 3C), which implies that microtubules loss would decrease the tangential/horizontal packing density of neuroepithelial cells. In these nocodazole-treated cerebral walls, the elastic modulus at the apical surface decreased in both mice and ferrets. This suggests that microtubules, which enable neural progenitors to apicobasally extend and are presumably under compression within each neural progenitor cell's process, may partly contribute to the overall stiffness of the apical surface. Since nocodazole treatment shrinks the morphology of neurons that are abundantly packed in the basal part of cerebral walls, it is also possible that cellular accumulation normally occurring in an expansive manner along the apicobasal axis partly contributes to the apical-surface stiffness. Notably, however, the elastic modulus decreased by a similar extent in both mice and ferrets, maintaining the original mouse<ferret relationship (*p* = 0.0002) (Figure 4).

These results suggest that although the stiffness on or near the apical surface of VZ depends on both actomyosin and microtubules, and the observed its mouse–ferret difference may not be sufficiently explained by the difference in dependence on these intracellular factors.

### Apical surface contractility is comparable between mice and ferrets

**Figure 5:**
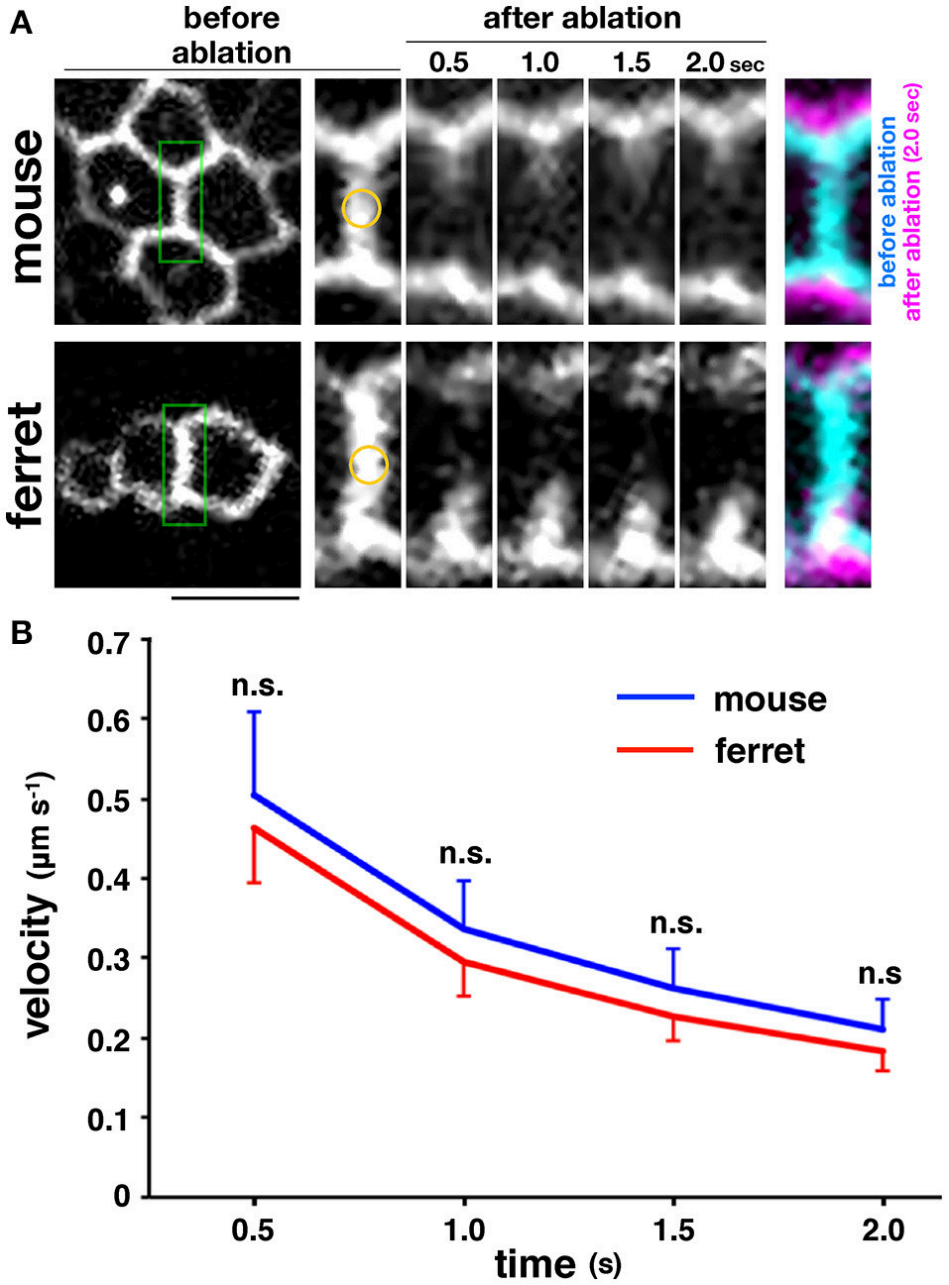
**Laser ablation experiments performed on the apical surface of embryonic cerebral walls**. **(A)** *En face* live observed EGFP-ZO-1^+^ endfeet of mouse and ferret VZ cells. A short-pulse laser was applied to the midpoint marked in yellow on the enlarged images of a boundary line enclosed in green formed by two polygonal apices of neighboring EGFP-ZO-1–labeled cells. The centrifugal separation of two vertices at both ends of the laser-targeted side were tracked for 2 s. In the final panels, images at 0 s and at 2 s are superimposed. Scale bar, 5 μm. **(B)** Graph depicting the velocity of time-dependent separation of the vertices tracked, showing that the separation velocity was comparable between mouse and ferret.

The myosin-blocking experiments described above recorded an almost similar reduction in the apical elastic modulus in the mouse and ferret VZs, which suggests that the actomyosin-dependent contractility of the apical surface may be almost identical in both species, and therefore may not contribute much to the overall mouse–ferret difference in apical surface stiffness. To further evaluate this possibility, we performed more direct stress-releasing experiments on the apical surface. Following the visualization of the apices of live VZ cells by transfection with EGFP-ZO-1 (Konno et al., 2008), we subjected *en face* inspected apical surfaces to laser ablation (Hara et al., 2013; Okamoto et al., 2013). We applied a short-pulse laser to the midpoint of a side (boundary line) formed by two polygonal apices of neighboring EGFP-ZO-1–labeled cells, and measured the separation of two vertices at both ends of the laser-targeted side (Figure 5A). We did not find significant differences between the velocity of separation of the tracked vertices of mouse (*n* = 33) and those of ferret (*n* = 37) (*p* = 0.80 at 0.5 s; *p* = 0.84 at 1.0 s; *p* = 0.73 at 1.5 s; *p* = 0.81 at 2.0 s, Mann-Whitney *U*-test) (Figure 5B). Hence we verified that the tangential contractility of apical surface is similar in both mice and ferrets.

### Adherens junction ultrastructure is similar in the VZ of both mouse and ferret

**Figure 6:**
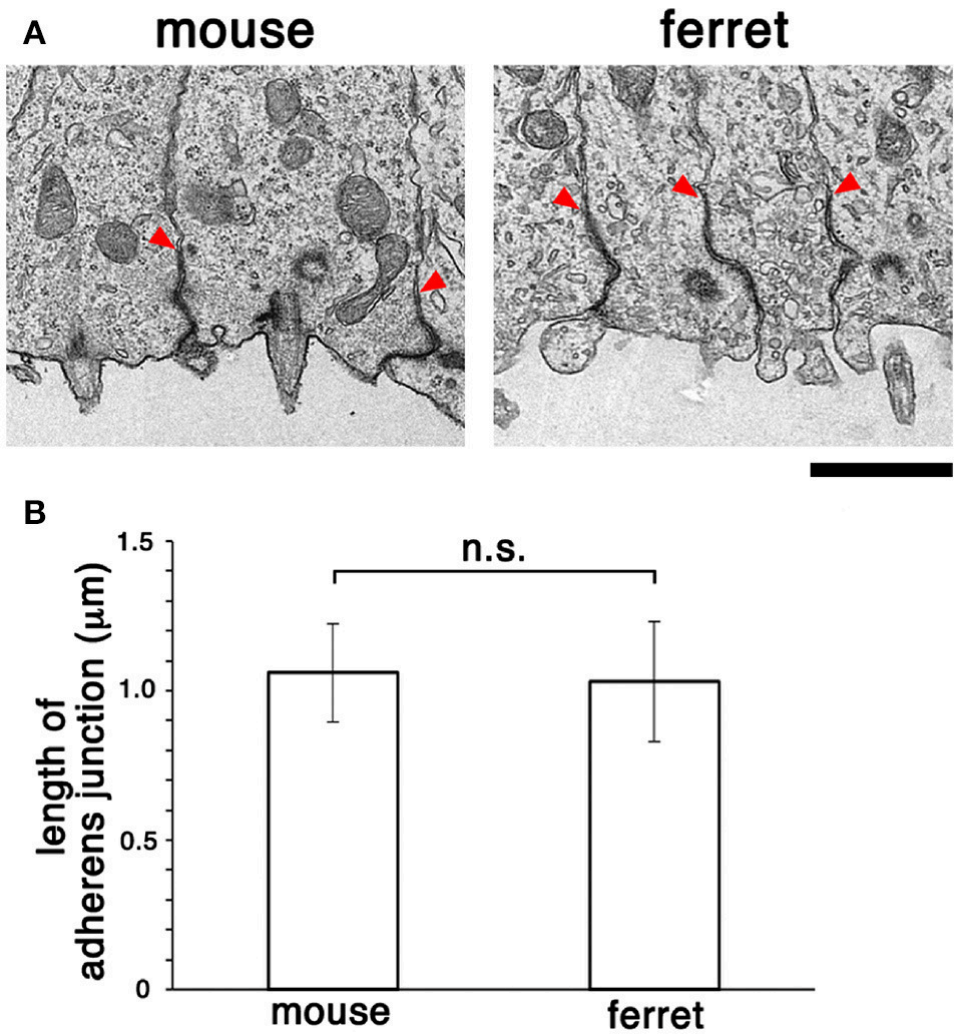
**Transmission electron micrographs (A)** showing cross-sectional views of adherens junctions, with basal borders indicated by arrowheads, in the VZ of mouse and ferret. **(B)** Graph depicting that the length of adherens junction was similar between the mouse VZ (*n* = 11) and ferret VZ (*n* = 12) (*p* = 0.50, Mann-Whitney *U*-test). Scale bar, 1 μm.

Following comparative *en face* inspection of immunostained adherens junction (Okamoto et al., 2014), which revealed a difference in the density of apical endfeet (i.e., difference in the horizontal size of each mesh, mouse < ferret), we sought to determine whether the adherens junctions in the mouse VZ and those in the ferret VZ are similar in their vertical size. Specifically, we sought to measure their length along the apicobasal axis using comparative transmission electron microscopy. As shown in Figure 6, the measured depth of the adherens junction was similar in both mice (1.06 ± 0.16 μm, *n* = 11) and ferrets (1.03 ± 0.20 μm, *n* = 12) (*p* = 0.50, Mann-Whitney *U*-test).

### Dissociated mouse VZ cells are stiffer than ferret VZ cells

**Figure 7:**
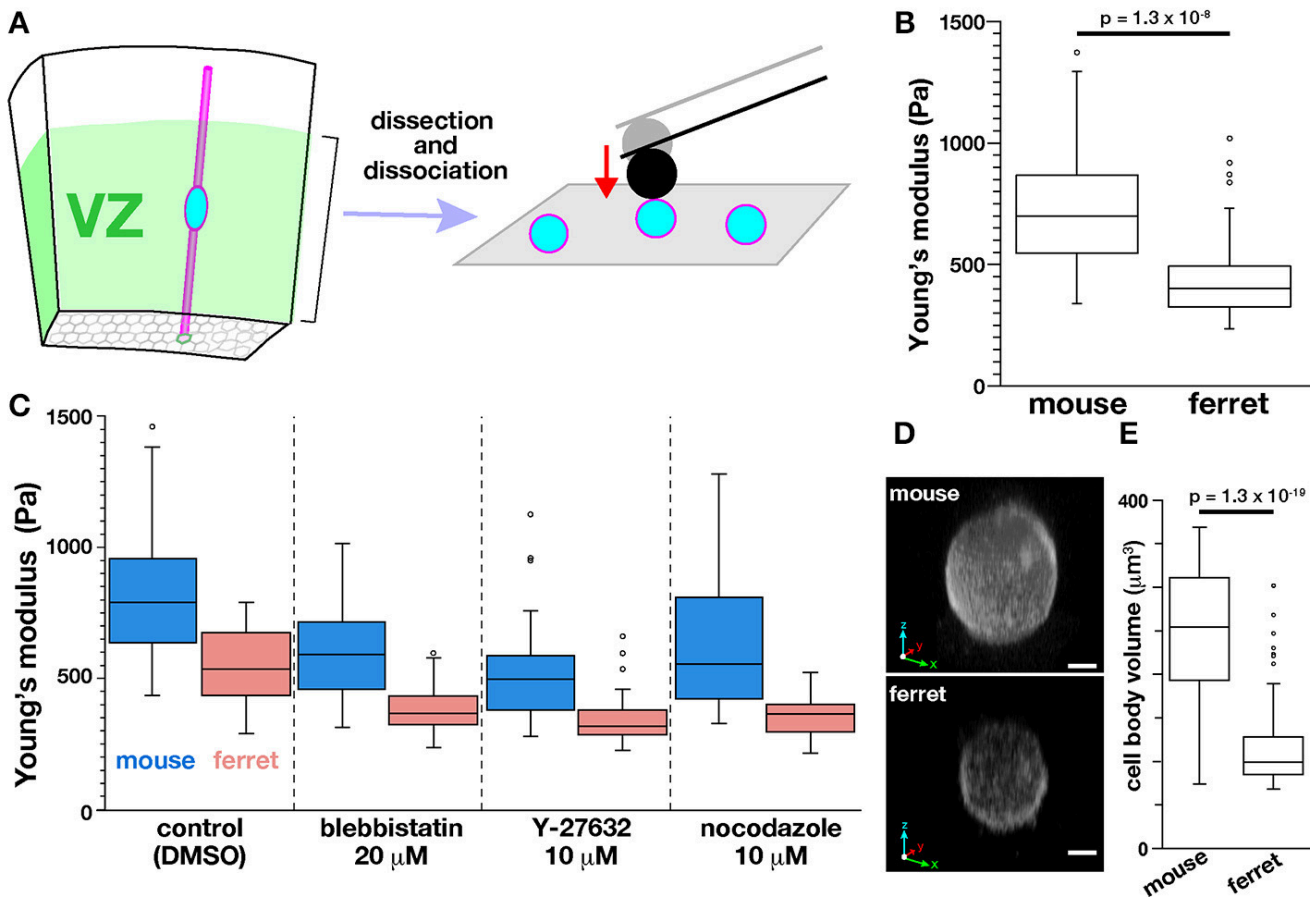
**AFM indentation measurements made on dissociated VZ cells. (A)** Experimental design. **(B)** Graph showing the elastic modulus of dissociated VZ cells in mice (*n* = 51) and in ferrets (*n* = 49) (*p* = 1.3 × 10^−8^, Mann-Whitney U-test). **(C)** AFM measurement made on dissociated cells under pharmacological treatments. **(D)** 3D reconstructed live images of FM4-64–labeled mouse and ferret VZ cells. Scale bar, 2 μm. **(E)** Graph comparing the volume of mouse and ferret VZ cells (*n* = 55 and 75, respectively, *p* = 1.3 × 10^−19^, Mann-Whitney *U*-test).

Next, to determine whether the tissue-level difference in stiffness on or near the apical surface between mice and ferrets can be explained by a possible difference in the stiffness of individual VZ cells of the two species, we performed AFM indentation measurements on single, dissociated cells. Most VZ cells were originally bipolar-shaped, and may have lost thin cellular processes, especially basal processes that were much longer than apical processes during microsurgical, enzymatic, and mechanical dissociation steps. Harvested cells were placed in culture dishes and pushed by a spherical bead (5 μm diameter) attached to the tip of a cantilever (with an indentation depth of 1 μm) (Figure 7A). Unexpectedly, the elastic modulus was significantly greater in mouse cells (718.7 ± 58.0 Pa, *n* = 51) than in ferret cells (452.7 ± 42.8 Pa, *n* = 49; *p* = 1.3 × 10^−8^, Mann-Whitney *U*-test) (Figure 7B). The values obtained for dissociated cells were both much smaller than those obtained for the apical surface of cerebral walls (1399 Pa in mice and 1734 Pa in ferrets). Pharmacological experiments as performed for cerebral walls (blebbistatin, Y-27632, and nocodazole) all reduced elastic moduli of dissociated cells, but interestingly, the mouse–ferret difference in single-cell stiffness (mouse > ferret in control, *p* = 5.4 × 10^−7^) was maintained even after inhibitor treatments (*p* = 1.4 × 10^−8^ in blebbistatin, *p* = 2.3 × 10^−6^ in Y-27632, and *p* = 2.8 × 10^−8^ in nocodazole) (Figure 7C).

Since LaPlace's law predicts that the pressure within a sealed bubble rises when its radius is reduced (Dai and Sheetz, 1999), we compared the sizes of mouse and ferret cells, expecting mouse cells to be smaller than ferret cells. Contrary to our expectation, the dissociated mouse cells (237 ± 72 μm^3^, *n* = 55) were significantly larger than dissociated ferret cells (116 ± 48 μm^3^, *n* = 75) (*p* = 1.3 × 10^−19^, Mann-Whitney *U*-test) (Figures 7D,E) for unknown reasons. These results suggest that the difference in stiffness of single dissociated cells (mouse > ferret) may not directly generate the differential tissue-level stiffness on or near the apical surface (mouse < ferret), and also that it may underlie or be associated with 3D cellular morphology/behaviors in the ferret VZ, namely denser horizontal assembly/packing, more slender nuclear and somal shapes, and a unique INM pattern.

### Processes are dominant over cell bodies subapically in ferrets compared with mice

Given that (1) tangential contractility of the apical surface was comparable between mice and ferrets (Figure 5), (2) dissociated ferret cells (which may represent cell bodies of ferret VZ cells) were softer than mouse cells (Figure 7), and (3) horizontal assembly of apical endfeet was denser in ferrets (Okamoto et al., 2014), we hypothesized that the tangential packing density of VZ cell processes, rather than cell bodies, at or near the apical surface, may be a major contributing factor to the tissue-level mouse–ferret difference in the stiffness on or near the apical surface of VZ.

**Figure 8:**
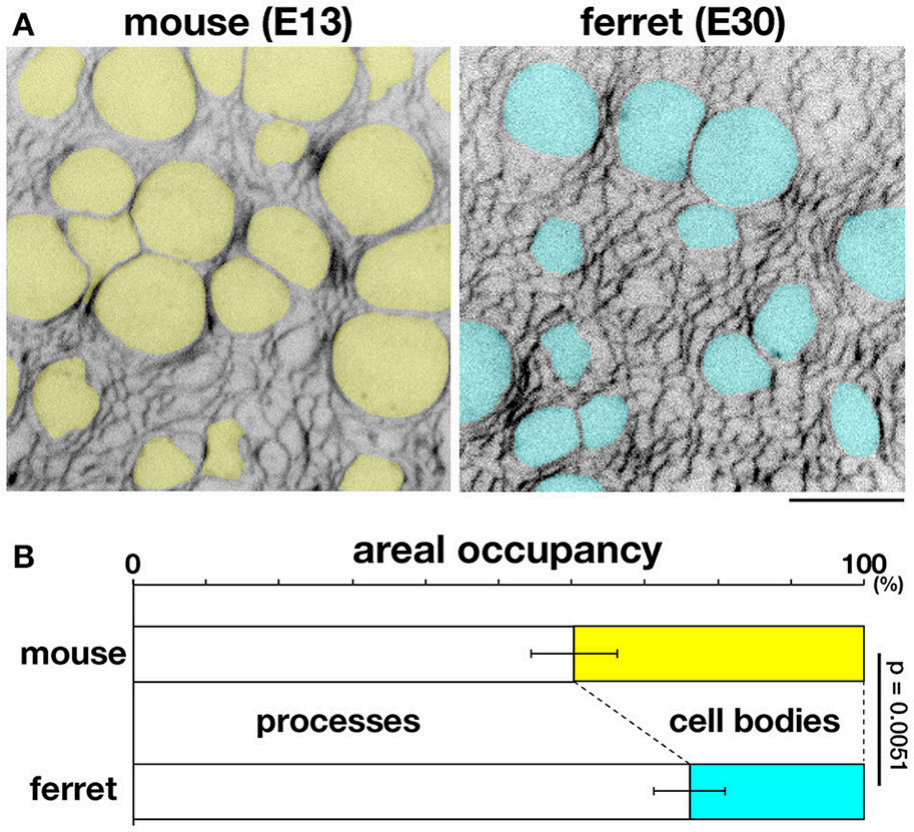
**Occupancies of the near-apical microzone by cellular processes and cell bodies**. **(A)** Horizontal confocal sectional live imaging at 5 μm from the apical surface of mouse and ferret VZ (FM4-64–labeled). Cell bodies > 3 μm in diameter at a depth of 5 μm, highlighted in yellow for mouse and blue for ferret, are surrounded by a mesh-like area composed of non-somal elements, i.e., apical processes. Scale bar, 10 μm. **(B)** Graph depicting the ratio of apical processes and cell bodies (*n* = 6, *p* = 0.0051, Mann-Whitney *U*-test).

To further investigate this possibility, we sought to measure the occupancy of apical processes and that of cell bodies in a microzone near (~5 μm from) the apical surface. Cell–cell borders were visualized throughout VZ by labeling plasma membrane with FM4-64 dye. In each horizontal section, which was completely filled with cellular elements and had no clear extracellular spaces, we examined the areal occupancy of either cell bodies (defined so if diameter of each sectional area was >3 μm, highlighted yellow or blue) or processes (seen as mesh) (Figure 8A).

At a depth of 5 μm, the occupancy by cell processes was significantly greater than that by cell bodies in both mice and ferrets (*p* = 0.0051, Mann-Whitney *U*-test): 39.7% for cell bodies and 60.3% for processes in mice [*n* = 6 (cerebral walls)]; 23.9% for cell bodies and 76.1% for processes in ferrets [*n* = 6 (cerebral walls)] (Figure 8B). At more superficial or apical levels (at 1–2 μm depths), the predominance of processes further increased, approaching >90% (data not shown). Since AFM force–distance curves are generally obtained under a much stronger contribution by the surface of a tested material than that by deeper parts, these results support our proposal that the tissue-level stiffness on or near the apical surface of VZ is best explained by the density of tangentially packed cellular processes, which is significantly greater in ferret (39.6 apices per 100 μm^2^) than in mouse (27.6 apices 100 μm^2^) (Okamoto et al., 2014).

### Stage-dependent decrease in density of endfeet/apices correlated with reduced apical-surface stiffness in mice

**Figure 9:**
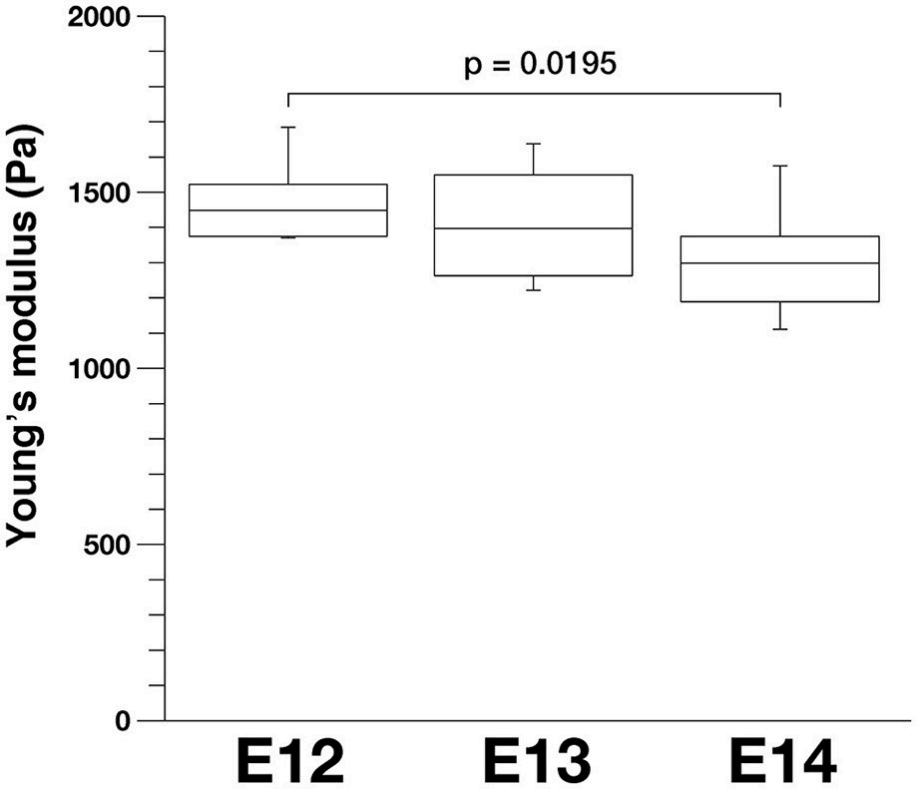
**Apical elastic modulus of the mouse VZ measured by AFM indentation at different embryonic days, ***n*** = 10 for E12, 12 for E13, and 15 for E14 (***p*** = 0.0195, Steel-Dwass test)**.

To further evaluate our model, we compared stage-dependent changes in the apical surface stiffness in mice and examined its possible correlation with changes in the density of apical endfeet. As shown in Figure 9, the elastic modulus measured at E14 (1327.4 ± 59.0 Pa, *n* = 15) was significantly smaller than that measured at E12 (1487.3 ± 57.8 Pa, *n* = 10) (*p* = 0.0195, Steel-Dwass test). Additionally, the elastic modulus measured at E13 was between those measured at E12 and E14, although not significantly smaller than at E12, and greater than at E14. We did not detect statistically significant differences between the elastic modulus of dissociated mouse VZ cells at E12 (734.6 ± 45.0 Pa, *n* = 64), E13 (718.7 ± 58.0 Pa, *n* = 51), and E14 (743.8 ± 72.8 Pa, *n* = 43). A previous study reported that the density of apical endfeet decreased during this E12-to-E14 period (35.7 per 100 μm^2^ at E12, 20.4 per 100 μm^2^ at E13, 12.0 per 100 μm^2^ at E14) (Nishizawa et al., 2007). Therefore, during this E12-to-E14 period, the elastic modulus obtained on or near the apical surface and the apical endfoot density both decreased, supporting the idea that horizontal cell-process densification may be a major factor that contributes to an increase in the vertical stiffness on or near the apical surface of the VZ.

## Discussion

The primary goal of this study was to determine if there is a measurable physical or mechanical difference between the mouse and ferret VZs. Our previous observation of differential INM behaviors between mice and ferrets (Okamoto et al., 2014) prompted us to speculate that VZ cells may sense and respond to mechanical factors in some way in order to alter their behavior. Motivated by the idea that the sensing of mechanical factors by VZ cells frequently occurs near the apical surface where multiple mechanical events, such as mitosis, resultant duplication of INM flow, and apical constriction, take place, we focused on a microzone close to (~10 μm from) the apical surface of the VZ. Through AFM indentation measurements, we found that the elastic modulus on or near the apical surface of the VZ was greater in ferrets than in mice. Additionally, inhibiting either actomyosin or microtubules partly reduced the apical stiffness of the VZ in both mice and ferrets and maintained the original mouse<ferret relationship. We suggest that the greater stiffness of the ferret apical surface compared with mice is largely explained by the denser lateral assembly of apical endfeet in ferrets. Such difference in stiffness may underlie differential INM behaviors between mice and ferrets.

### Lateral densification of VZ cells' apical processes increases stiffness in the vertical direction at the apical surface

Previous studies have shown that epithelial cells' densification can lead to a variety of cellular responses such as cessation of proliferation and epithelial-to-mesenchymal transitions (detachment from the apical surface) (Halder et al., 2012; Mariani et al., 2012; Mammoto et al., 2013). Assessing the mechanical properties of three-dimensionally developing tissues and microzones is therefore necessary in order to dissect underlying molecular mechanisms that may regulate how cells sense and transduce mechanical stimuli, as well as how they subsequently exhibit outcome behaviors such as proliferation and migration (Heisenberg and Bellaïche, 2013; Mammoto et al., 2013; Iskratsch et al., 2014). A recently reported method of oil droplet–mediated measurement of endogenous mechanical stress in tissues (Campàs et al., 2014) can be an ideal solution, but it also has technical limitations when thin structures such as cellular monolayers are subjected (Campàs, 2016). Preliminary trials for introducing oil droplets into the microzone ~10 μm from the apical surface of the VZ have not succeeded thus far due to its width and proximity to the surface (T. Shinoda, unpublished). As an alternative, we sought to instead measure the elastic modulus of the apical surface of the VZ.

Before beginning this study, we hypothesized that there is a mechanical link between the tangential density of subapically assembled cellular elements (cell bodies and/or processes) and the configuration of the inner/apical surface of the VZ, based on the following past experiences. First, we had previously observed that focally formed subapical overcrowding due to acute removal of basal processes from VZ cells resulted in apical bulging of that portion. This bulging caused the apical surface contour to become convex in contrast to the original/normal concave line (Okamoto et al., 2013). Second, mechanical simulation using a vertex model, where the volumetric energy within an epithelial-like cell is tightly associated with the apical elastic energy, successfully reproduced physiological and pathological aspects of embryonic mouse cerebral walls' behaviors (Okamoto et al., 2013). Since we found that the apical surface of the ferret VZ was denser than that of the mouse VZ (Okamoto et al., 2014), we expected that if AFM indentation measurement was performed vertically on the apical surface of the ferret VZ, it might yield a different elastic modulus than for mice.

Why is the apical surface vertically stiffer in ferrets than in mice both before (Figure 2) and after pharmacological experiments (Figure 4), and in a stage-dependent manner in mice (Figure 9) when it was tangentially denser in terms of assembled apical endfeet of VZ cells? Assembled apical endfeet form honeycomb structures with cell–cell junctions (adherens junctions) and underlying cytoskeletons. The ultrastructure of adherens junctions are similar in both mice and ferrets (Figure 6). We therefore speculate that denser existence of endfeet within the microzone containing the apical surface makes such mesh/honeycomb finer, thereby increasing mechanical resistance against a vertically applied force. It would be important to further compare cell-to-cell adhesiveness between the mouse and ferret VZ. Related to this first model, it is also possible that the apical-most part of the VZ cells' processes, ~5 μm from the surface, corresponding to the indentation depth in this study, may be more laterally compressed against each other in ferrets than in mice. This could therefore increase the local intracellular or intra-process pressure. Since our laser ablation experiment did not detect significant differences in the velocity of the vertices we tracked at both ends of the laser ablated side between mice and ferrets (Figure 5), the results do not directly support this second model. However, if the difference in cellular stiffness measured for dissociated mouse and ferret cells (Figure 7) may have affected the recoil behavior of laser-ablated apical-surface elements, the potential compression along the tangential and/or apicobasal directions in the apicalmost ferret VZ could have been partially masked.

Mechanical stimuli presumably sensed at or near the apical surface would function, both in mice and ferrets depending on its degree, as a trigger for activating intracellular signal transduction pathways in VZ cells to potentially regulate their nuclear and somal migration, and/or cell-cycle progression, which could lead to slower nuclear and somal movement in apical-ward G2-phase cells in the ferret VZ, and/or faster nucleokinesis in basalward G1-phase cells (Okamoto et al., 2014). In addition to such a possible cell-intrinsic mechanism, meaning that a VZ cell that senses the tissue-level apical-surface stiffness may regulate its nucleokinesis in an autonomous manner, non-autonomous mechanisms may also exist: VZ cell nuclei and somata that are approaching the apical surface during G2 or those that leave the apical surface during early G1 may be passively influenced (Kosodo, 2012; Lee and Norden, 2013) by tissue-level stiffness at or near the apical surface.

### Does cell body stiffness affect collective INM in crowded neuroepithelia?

Although a densification-induced lateral compression could be a causal mechanical cue for VZ cells in mice and in ferrets to behave differently, other factors may also exist. Despite a stage-dependent decrease in the density of apices in mice (E12 > E14), VZ cells' INM behaviors do not dramatically change between E12 and E14 (Ochiai et al., 2009; Okamoto et al., 2013). The lower stiffness of dissociated ferret VZ cells compared to mouse VZ cells (Figure 7) may contribute to the more slender nuclear and somal shapes of ferret VZ cells compared with mouse VZ cells *in vivo* (Okamoto et al., 2014). Therefore, the lower stiffness may also be associated with quicker basalward nucleokinesis in ferret VZ cells during G1. The mechanisms underlying the mouse–ferret difference in stiffness as well as the nuclear and somal sizes of dissociated cells are currently unknown; however, future measurements of the expression of molecules implicated in regulating nuclear shape and stiffness, such as those associated with the cytoskeleton and/or the nuclear membrane (Nagayama et al., 2014, 2015) in mice and in ferrets may provide useful information.

Our present finding regarding the stiffness, as well as the nuclear and somal sizes of VZ cells is also valuable for comparing the virtual neuroepithelium or the VZ of mice and ferrets. To better understand how INM behaviors of different VZ cells can be coordinated to achieve an ordered pseudostratified structure, we are trying to reproduce the movements of all VZ cells *in silico* by developing new mathematical modeling methods (Shinoda et al., unpublished). Adjusting parameters such as stiffness, as well as nuclear and somal sizes, based on experimental measurements would improve our simulations, which will contribute to fully understanding how neuroepithelium or VZ works as efficiently as possible in collective nuclear and somal traffic to maximize its productivity.

## Materials and methods

### Animals

To investigate the differences between the ferret and mouse neocortical VZ, we chose a mid-embryonic period during which layer V and layer VI neurons are commonly generated in a variety of mammalian species (Clancy et al., 2001), namely E13 in mice and E28–30 in ferrets as previously described (Okamoto et al., 2014). Pregnant ferrets were obtained from Marshall BioResources while pregnant ICR mice were obtained from SLC. For live visualization of the apical-surface mesh comprised by VZ cells' endfeet, we used a transgenic mouse line (R26-ZO1-EGFP: Accession no. CDB0260K; http://www2.clst.riken.jp/arg/reporter_mice.html) that ubiquitously expresses EGFP fused to mouse ZO-1 under the control of the *ROSA26* locus (Katsunuma et al., 2016). All protocols for animal experiments were approved by the Animal Care and Use Committee of Nagoya University.

### Preparation of cerebral walls for AFM measurements at the apical surface of VZ

Cerebral hemispheric walls (apicobasally 200–300 μm in mice and 300–400 μm in ferrets) were freshly isolated from embryos, processed (horizontally 400–500 μm × 400–500 μm, 1–2 pieces from a hemisphere) microsurgically in DMEM/F12, and transferred, with 1 ml of DMEM/F12, to a 35 mm dish previously covered partly with AteloCell IAC-30 collagen gel (Koken) at a concentration of 0.3 mg/ml. Cerebral walls were gently placed with their apical surface facing up on top of the gel portion (Figure 2B), which was approximately 5 mm thick and 20 mm in diameter (encircled by a silicone rubber ring attached to dish surface). The gel and cerebral walls were completely submerged in DMEM/F12.

### Preparation of dissociated VZ cells for AFM measurements and live imaging

Cerebral wall slices (coronal) were microsurgically divided into an inner portion corresponding to the VZ and the remaining outer portion. The inner portion (VZ) was treated with trypsin-EDTA (0.05%) (Thermo Fisher Scientific). VZ cells were dissociated by gentle pipetting (Figure 7A). Although the majority of the VZ cells were originally bipolar in shape and highly elongated (>100 μm long), the dissociation steps may have removed most of their long processes. It is possible, however, that mouse VZ cells that were stiffer than ferret VZ cells were more resistant to mechanical tearing of their processes. AFM measurements and acquisition of live images were started 30 min after plating of cells and finished within 60 min while cells were still rounded up with no spreading on the dish surface.

### AFM indentation measurement

All measurements were made with a Cellhesion200 (JPK Instruments) mounted on an IX71 inverted microscope (Olympus), which is equipped with a cantilever with a borosilicate bead (sQUBE, 20 μm diameter for the tissue-level measurement and 5 μm diameter for dissociated cells) (Figures 2B, 7A). The spring constant of each cantilever was determined before measurements were made using the thermal noise method (Hutter and Bechhoefer, 1993) in air (nominal value, 0.2 N/m). The applied forces were 10 nN for the cerebral walls and 1 nN for the VZ cells. The approach and retraction velocities measured were 5 μm/s for the cerebral walls and 1 μm/s for the VZ cells. Each measurement point was set in the central region of the apical surface of a cerebral wall or at the top of each dissociated VZ cell. Force–distance curves (Figure 2B) were acquired using the contact mode. Analyses of the obtained force–distance curves were performed with the JPK DP software v.5 (JPK Instruments). Briefly, the Hertz model (Hertz, 1881; Crick and Yin, 2007; Kuznetsova et al., 2007) was applied to calculate Young's modulus as follows:

$$\begin{array}{l} F=\;\frac{E}{1-\nu^{2}}\left[\frac{a^{2}+R^{2}}{2}ln\frac{R+a}{R-a}-aR\right] \end{array}$$

where *F* is the force, *E* is the Young's modulus, ν is the Poisson's ratio, *a* is the radius of contact circle, and *R* is the radius of sphere. The Poisson's ratio was set at 0.5 as previously described (Iwashita et al., 2014). The indentation depth was ~5 μm for the apical surface of the VZ and ~1 μm for the dissociated cells.

Although previous studies have reported measurements of the stiffness of different parts of neurons and glia attached on dishes (Lu et al., 2006), the original bipolar morphology of the VZ cells could not be reproduced in 2D culture. Therefore, we could not perform such cell region–specific AFM measurement. We attempted to measure the elastic modulus of the subapical zone of cross-sectionally prepared cerebral wall slices in which cell layers along the apicobasal axis can be seen, as previously described (Iwashita et al., 2014), but could not obtain reliable force–distance curves in the subapical microzone (~10 μm from the apical surface) that we focused on in a reproducible manner, probably due to its proximity to the surface. It should also be noted that the spring constant of the cantilever was 0.2 N/m in our study while it was 0.03 N/m in Iwashita et al. (2014).

### Pharmacological experiments

Cerebral wall pieces were treated with DMEM/F12 containing either 1% DMSO (Sigma) (11 pieces from 4 mouse embryos and 9 pieces from 3 ferret embryos), 20 μM blebbistatin (Calbiochem) (14 pieces from 5 mouse embryos and 11 pieces from 3 ferret embryos), 10 μM Y-27632 (Wako) (14 pieces from 5 mouse embryos and 10 pieces from 3 ferret embryos), or 10 μM nocodazole (Wako) (11 pieces from 4 mouse embryos and 9 pieces from 3 ferret embryos) for 30 min before being subject to AFM measurement. Some of the treated mouse cerebral walls were imaged for assessing bending/curling (Figure 3A), thinning coupled with lateral expansion (Figure 3B), or apical endfeet enlargement (Figure 3C) at the indicated timepoints. We examined whether these pharmacological treatments (30 min) affected the density of M-phase cells at the apical surface. While the density of cells positive for phosphohistone-H3 (pH3) was comparable (about 5.8 per 100 μm of the apical surface in coronal cerebral wall sections) between DMSO-, blebbistatin-, or Y-27632–treated E13 mouse hemispheres, it was a little smaller (about 4.2) in nocodazole-treated hemispheres, partly reflecting that occurrence of M-phase (arrival of G2-phase cells) at the apical surface is dependent on microtubules (Tsai et al., 2005; Xie et al., 2007). Based on the lateral expansion of nocodazole-treated cerebral walls (1.2~1.3-times longer dorsoventrally at the apical surface, Figures 3A,B), we obtained a corrected density for pH3^+^ cells at 5~5.5. Together, increase of M-phase cells (which would occur when mitosis is arrested) was not observed at the apical surface.

Dissociated cells were treated with DMEM/F12 containing either 1% DMSO (Sigma) (50 mouse cells and 38 ferret cells), 2 μM blebbistatin (43 mouse cells and 36 ferret cells), 1 μM Y-27632 (44 mouse cells and 33 ferret cells), or 1 μM nocodazole (38 mouse cells and 32 ferret cells) for 30 min before being subject to AFM measurement.

### Laser ablation of VZ cells' apices labeled with EGFP-ZO-1

Mouse ZO-1 cDNA fused with EGFP was subcloned from pCAG-EGFP-ZO-1 (gift from F. Matsuzaki) (Konno et al., 2008) into pEFX-LPL (Okamoto et al., 2013; Sakakibara et al., 2014). Sporadic visualization for apices of neural progenitor cells was achieved by transfection with a mixture of conditional expression plasmids, 0.5 μg/μl pEFX-LPL-EGFP-ZO-1 and 0.01 μg/μl pEFX-Cre. Transfection by *in utero* electroporation was performed using pregnant ICR mice as described previously (Okamoto et al., 2013). Briefly, the plasmid solution containing Fast Green was injected into the lateral ventricle of each E12 embryo. 33 V electric pulses were applied four times with forceps-type electrodes (CUY650P3, NEPAGENE). *Exo utero* electroporation was performed using pregnant ferrets as described previously (Okamoto et al., 2014). Briefly, plasmid solution containing Fast Green was injected into the lateral ventricle of E28 embryos, and 55 V electric pulses were applied four times with forceps-type electrodes. For ablation on the apical surface (Figure 5A), cerebral walls prepared at E13 (mouse) or E29 (ferret) were mounted apical-side down in a glass-bottomed 35 mm dish using collagen gel. Laser ablation was performed as described previously (Hara et al., 2013) with an IX81 inverted microscope (Olympus) equipped with CSU-X1 (Yokogawa), iXon3 897 EMCCD camera (Andor), 60x objective lens (UPLSAPO 60XW, Olympus), on-stage culture chamber (Tokai Hit), and MicroPoint (Andor) operated with iQ2 live cell imaging software (Andor). The culture chamber was filled with 95% O_2_ and 5% CO_2_. With image acquisition in 0.5-s intervals, a pulse of 365 nm laser illumination at 16 Hz was simultaneously applied to the cell boundary at the apical surface.

### Imaging of live cerebral walls and dissociated cells

Low-magnification time-lapse monitoring (Figures 3A,B) was performed as described previously (Miyata et al., 2001, 2004; Okamoto et al., 2013). For sparse labeling of neural progenitor cells, cerebral walls were treated with DMEM/F12 containing extremely fine crystals of 1,1-dioctadecyl-3,3,3,3-tetramethylindocarbocyanine perchlorate, DiI C18(3) (D-282; Molecular Probes) for 1–2 min at room temperature. This DiI suspension was made by adding 1 ml of DiI stock solution (10 mg/ml in ethanol) to 10 ml DMEM/F12. DiI-labeled cerebral walls were then coronally sliced, and embedded in AteloCell IAC-30 collagen gel at a concentration of 0.25–0.3 mg/ml. Slices were imaged using an Olympus IX71 [4x (Figure 3A) or 20x (Figure 3B)] equipped with an Orca ER camera (Hamamatsu Photonics). To measure the volume of each dissociated cell (Figure 6), cells were plated on polyethyleneimine-coated glass-bottomed 35 mm dishes (Iwaki) at a density of 5 × 10^4^ / cm^2^, and then stained with the styryl dye FM4-64 (Thermo Fisher Scientific) at a concentration of 5 μg/ml for 30 min (Kawaue et al., 2014).

For FM4-64–based 3D imaging of mouse VZ cells to be volume-measured (Figure 7), we harvested live VZ cells using Fucci mice, in which differentiated (cell cycle-exited) neurons in the basal region of the cerebral walls are most intensely positive for mKO2-hCdt1, while G1-phase cells in VZ are only weakly positive for mKO2-hCdt1 (Sakaue-Sawano et al., 2008). In our conventional epifluorescence imaging system (an Olympus IX71 equipped with an Orca ER camera), monitoring of mKO2-hCdt1 expressed by G1-phase VZ cells required a very high level of image intensification, which always resulted in over-intensification (image saturation) for neurons. Thus, such clear difference in mKO2-hCdt1 intensity enabled us to easily distinguish cell cycle-exited neurons and cell-cycling VZ cells including G1-phase cells. We discarded cells that were strongly mKO2-hCdt1-positive (i.e., neurons). The remaining fraction was composed of both cells completely negative for mKO2-hCdt1 expression (S, G2, and M-phase cells) and those showing only very weak mKO2-hCdt1 fluorescence (G1-phase cells). It corresponded to the cell fraction separately obtained from microsurgically harvested VZs (immunocytochemically >90% Sox2-positive). Volume-measured (FM4-64–stained) mouse and ferret cells' VZ identity was confirmed by anti-Sox2 immunostaining immediately after live analysis.

Live confocal images of these stained cells were obtained with an FV1000 laser scanning confocal microscope (Olympus) equipped with a 100x objective lens (UPLSAPO 100XO, Olympus) and an on-stage culture chamber (Tokai Hit) filled with 95% O_2_ and 5% CO_2_. Confocal images were processed and analyzed with ImageJ. To calculate cell volume, cell diameters were measured along the XY plane and volumes were estimated assuming that the cells form true spheres. Reconstructed 3D images of the dissociated cells were obtained using Volocity (PerkinElmer).

For confocal imaging of all VZ cells in the subapical space (Figure 8), processed cerebral hemispheric walls (500 × 500 μm) were stained with 5 μg/ml FM4-64 for 30 min (Kawaue et al., 2014) before being mounted on polystyrene cell culture dish (Corning) using AteloCell IAC-30 collagen gel (Koken). Live confocal images were taken with a BX51W1 microscope (Olympus) equipped with a CSU-X1 laser scanning confocal unit (Yokogawa), a 100x objective lens (LUMPLFL 100XW, Olympus), an iXon+ EMCCD camera (Andor), and an on-stage culture chamber (Tokai Hit) filled with 45% N_2_, 40% O_2_, and 5% CO_2_. Similar preparations were made for cerebral walls prepared from R26-ZO1-EGFP mice (Figure 3C).

### Immunofluorescence

To visualize the apical junctional meshwork (Figure 1C), cerebral walls were fixed in 4% paraformaldehyde prepared in phosphate buffer (pH 7.4) and subject to whole-mount staining with anti–ZO-1 mouse monoclonal antibody (33-9100, Thermo Fisher Scientific), followed by Alexa Fluor 488–labeled anti–mouse IgG antibody (A-11029, Thermo Fisher Scientific). To determine the VZ identity of dissociated cells subjected to AFM measurement or live-cell imaging for volume measurement (Figure 7), they were fixed with 4% paraformaldehyde in phosphate buffer (pH 7.4) and immunostained with anti-Sox2 rabbit polyclonal antibody (ab97959, Abcam), followed by Alexa Fluor 488–labeled anti–rabbit IgG antibody (A-11008, Thermo Fisher Scientific). To determine the density of M-phase cells at the apical surface, cerebral walls treated with DMSO, blebbistatin, Y-27632, or nocodazole were coronally frozen-sectioned (16 μm) and immunostained with anti-pH3 (rabbit, 06-570, MILLIPORE, 1:300).

### Electron microscopy

For scanning electron microscopy (Figure 1), cerebral hemispheres were fixed in 4% paraformaldehyde in phosphate buffer (pH 7.4), and subsequently in 2.5% glutaraldehyde in phosphate buffer (pH 7.4). The brains were further post-fixed overnight in 2.5% glutaraldehyde at 4°C, followed by 1% OsO4 in PBS and dehydration. The samples were trimmed and coated with osmium and examined under an S-800S SEM (Hitachi). For transmission electron microscopy (Figure 6), cerebral hemispheres were fixed with 2% paraformaldehyde and 2.5% glutaraldehyde in cacodylate buffer (pH 7.4), post-fixed with 2% OsO4 in cacodylate buffer, dehydrated, and embedded in Epon 812. Thin sections were mounted on formvar film–coated single-slot copper grids, stained with lead citrate, and examined under a JEM-1400Plus electron microscope (JEOL). We used ImageJ to faithfully measure the length of adherens junctions in each cross-sectional electron micrograph. We traced the entire length of a heavily electron-dense portion (usually started from the apical-most level and ran non-straightly).

## Author contributions

AN performed all AFM measurements and pharmacological experiments, and wrote the manuscript. TS carried out 3D imaging and wrote the manuscript. TK performed electronmicroscopy. MS and NU assisted laser ablation experiments. KN and TMa supported AFM measurement. AK supervised the experiments and wrote the manuscript. TMi designed the project, performed DiI-based imaging, and wrote the manuscript.

## Funding

This work was supported by a Grant-in-Aid for Scientific Research on Innovative Areas, “Cross-talk between moving cells and microenvironment as a basis of emerging order” 22111006 (TMi), from the Ministry of Education, Culture, Sports, Science and Technology of Japan, and 16H02457 (TMi) from Japan Society for the Promotion of Science.

### Conflict of interest statement

The authors declare that the research was conducted in the absence of any commercial or financial relationships that could be construed as a potential conflict of interest. The reviewer TS declared a shared affiliation, though no other collaboration, with several of the authors AN, TS, TK, KN, TMa, AK, TMi to the handling Editor, who ensured that the process nevertheless met the standards of a fair and objective review.
